# Type 1 *Diabetes Mellitus*: A Review on Advances and Challenges in Creating Insulin Producing Devices

**DOI:** 10.3390/mi14010151

**Published:** 2023-01-06

**Authors:** Sonia M. Rodrigues Oliveira, António Rebocho, Ehsan Ahmadpour, Veeranoot Nissapatorn, Maria de Lourdes Pereira

**Affiliations:** 1 HMRI-Hunter Medical Research Institute, New Lambton, NSW 2305, Australia; 2 CICECO-Aveiro Institute of Materials, University of Aveiro, 3810-193 Aveiro, Portugal; 3 Department of Biology, University of Aveiro, 3810-193 Aveiro, Portugal; 4 Drug Applied Research Center, Department of Parasitology and Mycology, Tabriz University of Medical Sciences, Tabriz 5166/15731, Iran; 5 Department of Parasitology and Mycology, Faculty of Medicine, Tabriz University of Medical Sciences, Tabriz 5166/15731, Iran; 6 Department of Medical Technology, School of Allied Health Sciences, Walailak University, Nakhon Si Thammarat 80160, Thailand; 7 School of Allied Health Sciences, Southeast Asia Water Team (SEAWater Team), World Union for Herbal Drug Discovery (WUHeDD), Research Excellence Center for Innovation and Health Products, Walailak University, Nakhon Si Thammarat 80160, Thailand; 8 Department of Medical Sciences, University of Aveiro, 3810-193 Aveiro, Portugal

**Keywords:** Type 1 diabetes, pancreas, organ-on-a-chip, stem cells, islets, 3D cell culture, micromachines, biosensors

## Abstract

Type 1 *diabetes mellitus* (T1DM) is the most common autoimmune chronic disease in young patients. It is caused by the destruction of pancreatic endocrine β-cells that produce insulin in specific areas of the pancreas, known as islets of Langerhans. As a result, the body becomes insulin deficient and hyperglycemic. Complications associated with diabetes are life-threatening and the current standard of care for T1DM consists still of insulin injections. Lifesaving, exogenous insulin replacement is a chronic and costly burden of care for diabetic patients. Alternative therapeutic options have been the focus in these fields. Advances in molecular biology technologies and in microfabrication have enabled promising new therapeutic options. For example, islet transplantation has emerged as an effective treatment to restore the normal regulation of blood glucose in patients with T1DM. However, this technique has been hampered by obstacles, such as limited islet availability, extensive islet apoptosis, and poor islet vascular engraftment. Many of these unsolved issues need to be addressed before a potential cure for T1DM can be a possibility. New technologies like organ-on-a-chip platforms (OoC), multiplexed assessment tools and emergent stem cell approaches promise to enhance therapeutic outcomes. This review will introduce the disorder of type 1 *diabetes mellitus*, an overview of advances and challenges in the areas of microfluidic devices, monitoring tools, and prominent use of stem cells, and how they can be linked together to create a viable model for the T1DM treatment. Microfluidic devices like OoC platforms can establish a crucial platform for pathophysiological and pharmacological studies as they recreate the pancreatic environment. Stem cell use opens the possibility to hypothetically generate a limitless number of functional pancreatic cells. Additionally, the integration of stem cells into OoC models may allow personalized or patient-specific therapies.

## 1. Introduction

*Diabetes mellitus* (DM) is the most common group of metabolic disorders affecting the population in 2021. More than one in ten people, which is equivalent to 537 million people worldwide, suffers from DM, making it one of the biggest health problems in the world [1]. It encloses a group of chronic disorders that can be split into four major categories: type 1 *diabetes mellitus* (T1DM), type 2 *diabetes mellitus* (T2DM), gestational *diabetes mellitus* (GDM) and monogenic diabetes. The first, type 1 DM, is characterized by dysregulated glucose metabolism, due to progressive autoimmune destruction of pancreatic beta-cells. The second, type 2 DM, results from beta-cell dysfunction combined with systemic insulin resistance. The third, gestational diabetes (GDM), occurs when the body cannot produce enough insulin or develops a glucose intolerance during pregnancy and, when not properly managed, it can evolve into a lifelong condition of T2DM [2]. Finally, monogenic diabetes, also called precision diabetes or maturity-onset diabetes of the young (MODY) and often mistaken for T1DM or T2DM, is a rare type of diabetes caused by changes or mutation in a single gene [3].

The physiological regulation of glucose metabolism in the human body is a feedback loop based on endocrine signaling between pancreas, liver, and glucose-consuming tissues. The signaling is assured by pancreatic hormones (insulin and glucagon) that are released into the blood stream. Endocrine cells are clustered together, thereby forming the so-called islets of Langerhans, which are small, island-like structures within the exocrine pancreatic tissue [4]. There are five cell types releasing different hormones: α-cells (producing glucagon); β-cells (insulin and C-peptide); γ-cells (pancreatic polypeptide; now known as pancreatic polypeptide cells (PP cells)); δ-cells (somatostatin) and ε-cells (ghrelin). Through these hormones, particularly glucagon and insulin, the pancreas maintains blood glucose levels within a very narrow range of 4–6 mM [4]. When the level of blood glucose rises, after food intake for example, pancreatic β-cells are stimulated to produce and release insulin which mediates the uptake of glucose, fatty acids, and amino acids in insulin-sensitive tissues. On the other hand, during sleep or in between meals, when blood glucose levels are low, glucagon is released from α-cells to promote hepatic glycogenolysis. The reduction of β-cell insulin production or defective responses to insulin in tissues are common characteristics of DM disorders and result in high blood glucose levels, termed hyperglycemia. In T1DM, insulin injections remain the “one-size-fits-all” treatment, but this option is not very effective and many patients (mainly children and adolescents) face severe complications. Research in T1DM continues to improve our understanding of this condition, allowing the development of novel prevention, diagnosis, and treatment options. Nonetheless, T1DM patients are highly heterogenous, with the disease arising from different etiologies, varied genetic backgrounds, and symptomatic at distinct stages and with different severities. Altogether, these suggest T1DM to be a condition best monitored and controlled via personalized medicine approaches. Moreover, our understanding of early onset of diabetes, its stratification, role of genetics, and environment (epigenetics) still requires improved research to enable pre-clinical detection and the most effective therapeutic options per patient. In this case, organoid, organ-on-a-chip, and stem cell research hold great promise and encompass novel approaches that can offer great advances and hopes for understanding and treating T1DM and associated disorders. This review explores T1DM with a particular focus on the emerging opportunities and challenges arising from new technologies in the fields of stem cell research and microphysiological in vitro models with the aim of developing personalized viable models for T1DM pre-clinical detection and management.

## 2. Type 1 *Diabetes Mellitus*

Type 1 *diabetes mellitus* (T1DM) incidence has been increasing by 2–5% worldwide with significant heterogeneity in this diagnosis by regions or continents [5,6]. Clinical care has significantly improved, raising quality of life and clinical outcomes for these patients, but more must be done to find a cure.

Type 1 *diabetes mellitus* (T1DM) is still the most common chronic autoimmune disease in young patients–diagnosed mainly in children and adolescents–and is characterized by the loss of pancreatic β cells; as a result, the body becomes insulin deficient and hyperglycemic, expressing a “classic” trio of symptoms: polydipsia, polyphagia and polyuria [7]. Due to this immediate need for exogenous insulin replacement, patients with T1DM require daily insulin injections due to the absolute insufficiency of endogenous insulin caused by the autoimmune destruction of pancreatic β cells. This therapeutic practice lasts a lifetime. The causes of these autoimmune responses are still unknown and commonly referred to as “environmental factors” that contribute to the development of the disease. Based on several studies and a comprehensive scientific effort, the current consensus within the scientific community is that the autoimmune response to β-cells is triggered by a set of different environmental factors in genetically predisposed individuals [8]. This process is mediated via the activation of autoreactive β-cell-specific helper CD4^+^ and cytotoxic CD8^+^ T-cells, which infiltrate the islets leading to apoptosis of β-cells [9].

### 2.1. Causes of T1DM

#### 2.1.1. Genetic Triggers of T1DM

The strong genetic contribution to T1DM is illustrated by the fact that siblings of a T1DM affected individual are 15 times more likely to develop the disease themselves when compared with individuals of the general population [10]. This translates in a cumulative increased risk of 6% till the age of 30–35 years, but the risk also increases when some susceptible genes are present [10,11]. Before the Genome-Wide Association Studies (GWAS) came up in the mid-2000s only six loci [12] were associated with the disease; now, after four decades of research, due to the contribution of GWAS, over 60 loci were uncovered as T1DM susceptible [13,14]. Concomitantly, the number of loci correlated with diabetes-associated disorders is expanding, and many seem population-specific, such as the two SNP (single nucleotide polymorphisms) loci STT3B and PALM2 [15].

It has been long established that much of the genetic risk associated with T1DM is conferred by the human leukocyte antigen (HLA) region on chromosome 6p21 [16]. HLA proteins present antigenic peptides for T cell immune surveillance. Genetic variation in the HLA genes influences the peptide pool that can be displayed and recognized to initiate an immune reaction [17]. Class I HLA molecules (HLA-A, HLA-B and HLA-C) present endogenous antigens to CD8^+^ (cytotoxic) T-cells, while class II HLA molecules (DP, DR and DQ)) present antigens to CD4^+^ (helper) T-cells [18].

The high frequency of single nucleotide variants (SNVs) in HLA genes results in a total of 35,220 classical HLA alleles in November 2022 (https://www.ebi.ac.uk/ipd/imgt/hla/about/statistics/ (accessed on 30 November 2022)). Alleles at the HLA DR and HLA DQ class II loci are the most useful determinants of inherited risk of T1DM, especially in genes that encode highly polymorphic β chains (DRB and DQB) [14]. Different non-HLA regions have been associated to T1DM, e.g., [19,20]; however, their contributions are weaker compared to HLA regions. For more on the prediction of T1DM through genetics please refer to [21,22,23].

#### 2.1.2. Environmental Triggers of T1DM

One of the pieces of evidence that T1DM is not an entirely genetic bounded disease is that the concordance of T1DM among monozygotic twins is only about 30 to 50% overall [24]. This suggests that an environmental context for T1DM is also important (Table 1).

Viral infections have caught attention as potential environmental triggers of T1DM. Some studies in animal models have demonstrated that infections might trigger islet autoimmunity via several distinct mechanisms [25], although it is important to note that the current scientific reports failed to demonstrate the direct causality between infections and T1DM in humans. However, indirect associations suggest a link between T1DM development and some human viruses including enteroviruses, herpesviruses, rotaviruses, retroviruses, and picornaviruses [26]. Enteroviruses (EVs) are the prime suspects of a potential T1DM trigger [27]. Additionally, some evidence show that the gut microbiome could protect from the development of T1DM by promoting intestinal homeostasis [25]. Different environmental risk factors for T1DM have also been reviewed elsewhere [28,29].

### 2.2. Mechanism of T1DM Autoimmunity

**Figure 1:**
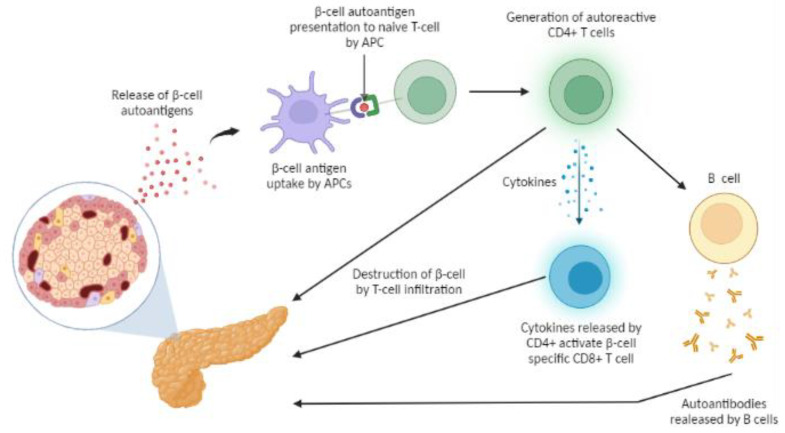
Overview of T1DM physiopathology. T1DM results from the autoimmune destruction of β cells of the islets of Langerhans in the pancreas. As β cells mass declines by T-cell-mediated apoptosis, insulin secretion decreases. To prevent autoimmunity, B lymphocyte cells must be silenced as they express autoreactive antigen receptors that interact with self-reactive T lymphocyte cells. Particularly important are CD4^+^ (helper) and CD8^+^ (cytotoxic) T cells. CD4 and CD8 are glycoproteins expressed in the membranes of some T cells (as well as macrophages, monocytes, dendritic cells and neutrophils). CD4 and CD8 positive cells recognise peptides present in the APCs, antigen-presenting cells. Created with BioRender.com.

One of the characteristics of T1DM is the recognition of β cell proteins as autoantigens by auto-reactive CD4^+^ and CD8^+^ T-helper cells and autoantibodies. Several autoantigens have been attributed to T1DM including insulin, glutamic acid decarboxylase 65-kDa (GAD65), islet antigen 2 (IA-2) [37], zinc transporter 8 (ZnT8), non-specific islet cell antibodies (ICAs), islet mitochondrial autoantigen imogen-38, pancreatic duodenal homeobox factor 1 (PDX1), chromogranin A (CHGA), islet-specific glucose-6-phosphatase catalytic subunit-related protein (IGRP), heat shock protein 60 (hsp60), and islet cell antigen 69 (ICA69) [38]. Figure 1 illustrates an overview of T1DM autoimmunity mechanisms.

It has been proposed that β-cell loss is caused by lymphocytic infiltration of the islet by dendritic cells, macrophages, and T lymphocytes. Autoreactive T lymphocyte cells specific for β-cell autoantigens, such as insulin, GAD65, IA-2, and ZnT8, have been identified [39]. It is difficult to pinpoint the main factor that triggers these autoreactive responses; nevertheless, it is well acknowledged that specific autoantigens are processed by Antigen-Presenting Cells (APCs). APCs include dendritic cells (DCs), macrophages, and β cells in the pancreatic islets. The autoantigens are then presented to naive T cells by “diabetes associated” HLA molecules to contribute to priming and expansion of pathogenic T cells and generation of autoreactive CD4^+^ T cells. These activated CD4^+^ T cells will then produce cytokines and, subsequently, activate beta-cell-specific cytotoxic CD8^+^ T cells. The activated T cells will migrate to pancreatic islets via vascularization and stimulate macrophages and other T cells, contributing to the destruction of islet β-cells [16]. Due to the decrease in β-cell number, the workload on the remaining β-cells is dramatically increased. This can induce apoptosis through a variety of pathways, such as the elevation of stress levels in the endoplasmic reticulum (ER), where misfolded or unfolded protein accumulates [40]. Some essential molecular mechanisms are yet to be clarified such as, for example, what is the exact role of autoantigen specific CD4^+^ T cell response, and if there is any primary autoantigen in T1DM, and if so, which one. Proinsulin or insulin has been proposed to function as the primary autoantigen [41,42], but so have GADA/GAD65 or ICA (islet cell autoantibodies) [43]. The true primary autoantigen (AAg) has not been definitively identified.

Understanding the nature and clinical utility of autoantigens is a central focus in diabetes research. Using AAgs as biomarkers has been shown as pivotal for prediction prior to disease onset and diagnosis–autoantibodies against β-cell proteins and peptides are now used almost routinely to predict the disease and help diagnose of T1DM [44]. Their recognition as biomarkers of pre-symptomatic disease has led to proposals for early type 1 diabetes staging using a range of autoantibodies for diagnosis–a concept that is starting to make its way into practice [45,46,47]—and it also important for the development of autoantigen-specific tolerance induction immunotherapy [16], such as for example targeting the ZnT8 antigen [48].

### 2.3. Treatment Options for Type 1 Diabetes

Diabetes left without proper treatment can cause a plethora of complications, many of which life-threatening. Acute complications include hypoglycemia, diabetic ketoacidosis, or hyperosmolar nonketotic coma (HHNC). Long-term complications include cardiovascular disease, diabetic nephropathy, and diabetic retinopathy [49]. Even though hyperglycemia could be controlled by drug administration or exogenous insulin, these treatments are unable to provide regulation of blood glucose. Ideally, a viable treatment for T1DM would restore both insulin production and secretion regulation by glucose. Currently, no real cure exists for diabetes, and daily insulin injections remain the standard of care for patients with T1DM. This treatment is lifesaving, but it conveys a chronic and costly burden of care, a persisting risk for acute and chronic complications, and it still results in an overall decreased life expectancy [50].

**Figure 2:**
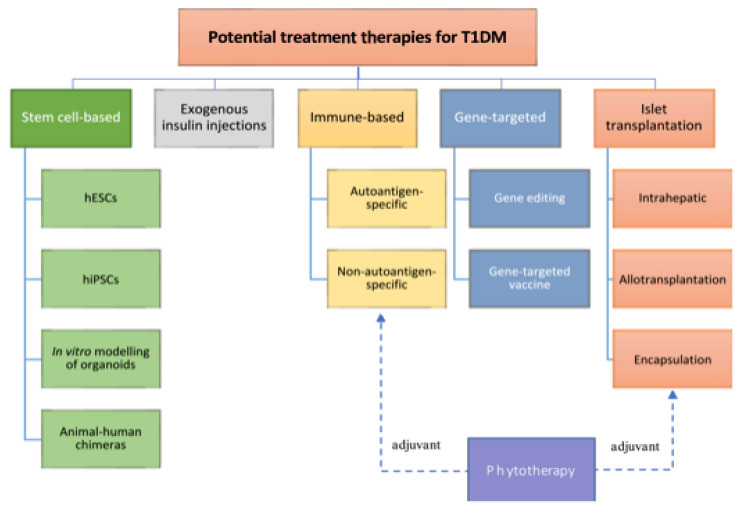
Current attempts to model a viable treatment option for T1DM. Insulin injections remain the standard treatment. Improvements in new insulin formulations, continuous insulin, and now coupled glucagon infusion pumps and continuous glucose monitoring systems represent advances in care, but are still cumbersome, imprecise, and costly [51]. Gene therapy has shown great promise as a potential therapeutic to treat T1DM, although its safety still needs to be confirmed in humans [52]. The success obtained in clinical studies regarding monoclonal anti-CD3 antibody in established T1DM demonstrates that modulation of islet autoimmunity in humans after the onset of overt disease can be achieved and gives some reason to be cautiously optimistic on their ability, and other immune-based therapies, to provide an effective treatment for the disease [53]. Islet transplantation is already established as an alternative therapy and is considered a relatively safe procedure with much lower associated risks compared to any other solid organ transplantation [54]. Stem cell-based approaches are the most promising methods for modulating a viable DM treatment model and can be combined with other techniques. Additionally, alternative approaches are investigating the use of natural products isolated from plants as adjuvants, either for encapsulation or immune-based T1DM therapies [55,56,57]. Islet Transplantation and Stem cell-based therapies will be further discussed in this review.

Figure 2 summarizes some of the most relevant approaches in DM clinical research. Although promising, alternative therapies to standard insulin injection are still a long way from clinical trials, and for now, patients still need lifelong treatment, and the relieving effect often only lasts for a few years. To improve therapy for T1DM patients and to develop new approaches, there is a strong need for further research towards the understanding and characterization of the factors leading to T1DM and its underlying mechanisms.

### 2.4. In Vivo Research on Diabetes Mellitus

Aiming at understanding the pathogenesis of the disease, animal models have been of particular interest, and more recently the designated humanized animal models [58], such as the YES mouse [59]. To reproduce faithfully the human immune response, it is desirable that animal models show spontaneous autoimmunity. A defining characteristic of T1DM is insulitis. This is predominately a lymphocytic infiltration of the islets of Langerhans targeting the β-cells and is seen in both humans and animal models, which spontaneously develop Type 1 diabetes [60]. Most experiments have been carried out in rodents. The most-used autoimmune models of type 1 diabetes are the non-obese diabetic (NOD) mouse [61,62] and the biobreeding (BB) rat [63], both first identified and bred in the 1980’s. Other popular animal models include the LETL (Long-Evans Tokushima Lean) rat, the KDP (Komeda diabetes-prone) rat and, the LEW-iddm rat [64,65], all models of spontaneous T1DM. A good comparison on T1DM animal models has been described by Mordes et al. (2004) and by Kottaisamy et al. (2021) [66,67].

Moreover, other transgenic and knock-out models of human genes have been established to study autoimmunity and transplantation “humanized models” [53,58,59]. Viruses have also been used to induce insulin-dependent diabetes (not autoimmune) in wild type mice. And diabetogenic agents like streptozotocin that have β-cell toxicity have been used to create chemically induced T1DM models [60,64,65,66,67].

These models allowed researchers to control and identify in vivo genetic and environmental factors that may affect diabetes onset and progression as well as its complications. Animal models for pharmacological testing are chosen with putative mechanisms of T1DM and newly developed drugs in mind. However, no promising therapies resulting from studies using mice have resulted in prevention or reversion of T1DM in the clinic. Succinctly, we next summarize the two most-used animal models.

#### 2.4.1. The Non-Obese Diabetic (NOD) Mouse

The most common T1DM model, the NOD mouse, is prone to spontaneously developing autoimmune diabetes, which mimics many features of human disease such as, islet infiltration by immune cells (insulitis) and the development of autoantibodies [61,68,69]. It has been subjected to numerous successful experimental interventions, a majority of which have later failed in human clinical trials [68]. Nonetheless, the number of similarities of human T1DM pathophysiology is astonishing, including similar polymorphisms that confer disease risk and the involvement of similar genes or biological pathways on the disease’s onset as well as immune response [68,70]. However, the translation of discoveries and therapies into humans has failed thus far. This highlighted the limitation that animal models have at mimicking human pathogenesis, hence the need for newer, more relevant models that mirror immune system and β-cell physiology.

#### 2.4.2. The Biobreeding (BB) Rat

The biobreeding (BB or BBDP) rat model for insulin-dependent *diabetes mellitus* was developed around the same time as the NOD mouse model. This spontaneously diabetic rat closely resembles characteristics observed in humans, being a good model to study genetic, immunological, and environmental components of the disease [71]. Interestingly, these rats have lower circulating osteocalcin on their littermates at onset of glycosuria, similarly to what is known to happen in children (for more on the effects of diabetes on bone biology please refer to [72], for example). This rat model has been extensively studied and described [73,74,75,76]. It is less used than the NOD mouse model, likely also due to the animals’ maintenance and use requirements in laboratory. However it offered valuable contributions to our current knowledge on T1DM. It has its particularities that set it apart from the NOD mouse model; for example, the incidence of diabetes is equal between both genders in the BB rat while NOD female mice have higher prevalence of the disease. For more, please refer to [77].

Both animal models offer advantages and disadvantages and are better suited for testing specific applications or approaches. For example, the BB rat due to its bigger size has advantages when testing certain injections or allo/xenografts and this includes allowing a routine collection of blood for continuous screening.

More animal models have been well documented by King and Bowe [60]. A recent review underlines the role of animal models for T1DM studies, namely those induced by diabetogenic agents such as streptozotocin and alloxan [67].

### 2.5. In Vitro Research on Diabetes Mellitus

Animal models continue to be a centerpiece in basic research and preclinical studies. Indeed, animal models provided significant clues on DM mechanisms. But although a variety of animal models for diabetes research have been developed and widely employed in mechanistic studies, the translation to humans has been mostly unsuccessful, thus suggesting the limited clinical relevance of the findings. Due to the complexity and the various factors involved in T1DM, as well as species-specific mechanisms, the identification and prediction of pathological pathways in humans is often not possible based on animal data–animal and human pathophysiology feature significant differences and have usually many limitations (e.g., animal size, availability, cost). Also, most animal models are developed to address one specific aspect of DM, and do not consider multiple other factors involved or the synergies between them. Due to this inability to recreate human diabetes in animal models (in vivo), there is a strong need for new and advanced in vitro models that enable the recapitulation of the complex physiology of the human body [78]. On the other hand, an endpoint for drug testing is too often the death of the animal. Here, the ongoing development of microfluidics and of organ-on-a-chip (OoC) models can offer novel options in biomedical research to tackle different stages of disease simulation and effective testing of drugs, including the most effective sequence of administration and drug combination. Based on advances in stem cell technology and tissue engineering, together with microfabrication, promising new approaches and systems have been introduced in recent years. We do not aim to discuss the different approaches to microfabrication nor pinpoint which state-of-art microfluidics would work best when combined with tissue bioengineering. Rather, we propose to reflect on the different aspects of these two novel and upcoming technologies and in their combined potential to aid T1DM research and eventually, clinical treatment.

#### 2.5.1. Requirements for the Establishment of In Vitro Models

The versatility of in vitro models gave unpaired insights into basic and translation research questions. It allowed homogeneity and reproducibility in data obtained. Novel in vitro techniques are contributing to make meaningful changes in modern medicine, particularly for diagnosis applications. These include microfabricated devices such as point-of-care diagnostics, biomedical microdevices, blood analogues in microdevices, biosensors and organ-on-chips. In the last example, the huge advantage lies in controlling cell microenvironments and maintaining tissue-specific functions to better mimic human physiology. They can resemble organoids in having multiple cell types, but they offer another layer of complexity by including mechanical cues and simulating the blood flow.

##### Cell Sources

**Figure 3:**
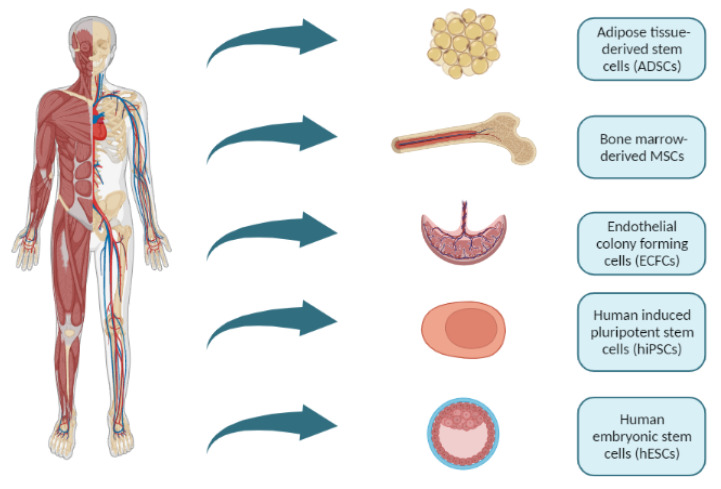
Sources of stem cells. Studies have shown that adipocytes and bone marrow are a good source of mesenchymal stem cells (MSCs). MSCs are part of adult stem cells (hASCs) present in the adult human body that are highly multipotent and capable of differentiating into several specialized cells. Endothelial colony forming cells (ECFCs) are vascular stem cells isolated from the mononuclear fraction of umbilical cord blood. Created with BioRender.com.

To mimic in vivo human physiology, it is essential to make an adequate choice of cell type and sources (Figure 3). A great tool used to study disease etiology at molecular levels is patient-specific cells. Primary cells, isolated from biopsies, for example, have been widely used in cancer research, where patient-derived tumors are cultured for drug screening purposes [79]. Still, primary human cells are typically only available in small quantities, which limits their usability in experimental set ups. This limited availability can be solved by derived cell lines from primary cells using specific genetic manipulation, but this procedure often has the downside of losing their specific phenotypes if continuously grown in culture.

Human stem cells have been of growing interest for in vitro models, disease modeling, and cell-based therapy, including human adult stem cells (also called somatic or tissue-specific stem cells) (hASCs), human embryonic stem cells (hESCs) and human-induced pluripotent stem cells (hiPSCs). Adult stem cells with better perspectives to be used as models of disease are neural progenitor stem cells (NPCs), mesenchymal stem cells (MSCs) and hematopoietic stem cells (HSCs) [80]. These are multipotent cells found in the adult body, where they are part of the regenerative process of specific tissues. Both hESCs and hiPSCs can differentiate into any kind of cell types in the body. Human embryonic stem cells are isolated from blastocysts while hiPSCs are patient cells reprogrammed to their pluripotent state. Human-induced pluripotent stem cells have the advantage of being patient-specific and their somatic origin.

The main limitation to the use of stem cells is the randomness associated with the differentiation process for certain cell types, which can lead to heterogenous populations of cells [81]. This leads to the need to establish standardized protocols and procedures for the differentiation process and appropriate cell functionality tests as well which, can further stress the cells.

Studying DM and its molecular drivers on human explants is difficult due to a limited access to relevant tissue samples. The stem cell technology, especially hiPSCs, enable research on patient-specific mechanisms on a molecular, biological, and cytological level [82]. The potential of these cell sources will be further discussed in Section 4 and Section 6. Their potential is immense and can be combined with other technologies such as microfluidics.

##### In Vitro or Ex Vivo Cell Culture Technology

Apart from an appropriate choice of the cell source, in vitro modeling requires culture technologies that provide a physiologically accurate microenvironment for the cells. Conventional 2D monolayer cultures have given valuable contributions to biomedical research and remain the most used method, but they have the major limitation of being isolated from their native microenvironment. More relevant physiological 2D models have been created such as culture system and cell patterning [82]. A 3D cell culture model which is sometimes called 3D engineering enables achieving higher complexities and more physiological relevance by promoting higher levels of cell differentiation and tissue organization. These were mainly possible by creating scaffolds to allow growth in all three directions. Popular types of scaffolds include hydrogels and inert matrices such as porous polystyrene membranes [83]. Some of these materials have been reviewed elsewhere [84]. Another prominent approach of 3D modeling is using organoid technology or scaffold-free 3D-cell culture methods, where cells self-assemble into clusters or spheroids. Organoids consist of 3D clusters of cells derived from primary or stem cell sources. They self-renew and self-organize into complex organ-like tissues, providing an outstanding potential to model human organ development [85,86]. Their potential is great and, for instance, just recently Romiti et al. successfully developed a transplantable human thyroid organoid from hESC [87].

Despite mimicking parts of complex 3D organization of organs, organoid models are limited in terms of emulating the entire functionality of the in vivo situation. Organoids often lack specialized cell types, mesenchymal compartment, microbiome and most importantly, vascularization [88], thereby still relying on traditional dish culture. Vascularization is essential to enable a physiological continuous transport of soluble factors (nutrients, oxygen, cytokines, hormones, drug compounds) to the tissue, as well as metabolic resultants (waste) away from the tissue [88]. Also, conventional 3D models most often still lack integration of mechano-physiological parameters and cannot be subjected to controlled tissue-level stimuli like shear stress, tension, and compression forces [88].

In the follow up of overcoming these limitations, microfluidic organ-on-a-chip technology (OoC) arises. In general, it promises to combine the advantages of cell culture (human background) and animal models (complex physiology). OoC is a microfluidic-based device engineered to mimic the physiology of an organ via culture and grow living cells and organoid substructures in a controlled micro-environment [89] that emulates crucial parts of physiological functions including mechano-physiological parameters, spatiotemporal chemical gradients, as well as vascular perfusion [60]. Basically, 3D cells are grown in scaffolds within a chamber or chambers of a microchip where small channels allow liquid flow (μL or pL volumes) that transport and distributes nutrients and other factors throughout the cells. Also, a feature of particular importance is the possibility of using analysis and imaging tools [60] that help monitor in real time the spatiotemporal organization of in vivo-like tissue architecture [62]. Those features are particularly important in drug development, toxicological screening, and disease modeling.

Many physiological functions and pathological conditions are not attributable to one specific organ but, rather, emerge from the interaction of multiple systems. It is in this context that the concept of multi-organ models takes particular interest, since organoids are not capable of modeling biological interactions at higher levels of organization (for example, tissue–tissue or multi-organ interactions) [60]. The field of organs-on-a-chip has laid the groundwork for engineering multi-organ in vitro models, yet a successful interconnection faces several challenges of conceptual (i.e., standardization and scaling), technical (i.e., tight seals and robust connectors) as well as biochemical nature (i.e., appropriate culture media composition) [63]. Moreover, an organoid-on-a-chip technology is also currently being considered, particularly for bioprinting and drug development [90].

## 3. Novel In Vitro Models–Microfluidic Technologies Applications in T1DM

Human stem cell technology combined with OoC platforms hold the promise of playing a relevant role in the understanding and treatment of T1DM. In fact, OoC models have already begin to play a part on drug development and preclinical safety testing, but their future holds promise in other areas as well. In this next part we will discuss viable models with the potential to identify treatment options for T1DM.

### 3.1. Pancreas-on-a-Chip

Pancreas-on-a-chip (PoC) focuses on the study of the endocrine part of the pancreas on a microfluidic chip. This enables the emulation of the function of in vivo human islets in a relatively equivalent in vitro environment [89]. Human PoC technology is a quick evolving platform for in vitro modeling of islet physiology and biochemistry that is now being considered for clinical islet transplantation.

A variety of microfluidic devices have been presented to recreate the microenvironment of the pancreas to study islet function. Different functional assays provided further information regarding the use of microfluidics as an excellent tool to perform comprehensive islet analysis and to obtain a more significant predictive value for islet functionality. The ability of having a high-sensitive tool for islet functionality analysis under oscillatory conditions may be of extremely value for diabetes research [91]. This demand led to the development of different approaches and PoC designs, many of which focusing on islets of Langerhans. Islet-on-a-chip (IOC) designs offer the possibility of continuous monitoring and evaluation of beta-cells function whether at single or multi-islet levels. Additionally, the advancement of microfluidic designs (for a review see [89]) and coupling of imaging-compatible biomaterials and biosensor technology is being tested as a tool to predict islet transplantation outcomes. With a step further of combining pancreatic islets with other tissue types, this may create a minimal, highly controlled in vitro environment to study diabetic interventions and test personalized therapies just prior clinical application with high rigor and efficiency.

### 3.2. Microfluidic Perfusion Systems for Pancreatic Islet Research

Microfluidic perfusion systems (MPS) designs allow them to assess media flow over single cells or cell culture chambers. This can simulate the in vivo-like microenvironment of pancreatic islets and multiple research groups have specialized in MPS developing [92]. These devices usually have two major components. The first is an islet-trapping mechanism, of immobilizing single or multiple islets, maintaining constant perfusion. The lack of perfusion in previous models presented a real limitation for various reasons that include the impossibility of dynamic monitorization in a static chamber, the increasing accumulation of secreted products in the microenvironment and the difficulty in getting long-term cell survival due to the lack of a continuous fresh flow of nutrients [92]. The lack of ‘perfusion’ can also be seen as a major problem in organoids. Some trapping mechanisms used in islet immobilization are dam wall-like or nozzle-like traps [93,94]. The second major component of islet MPS is an assessment tool to monitor islets glucose-dependent responses. The main techniques that have been successful in incorporating MPS are the capillary electrophoresis immunoassay (CEI) and the monitoring of intracellular Ca^2+^ oscillation [92]. CEI provides a technique for the direct detection of islet secretion with a detection limit of 3 nM for insulin [95]. Using this technique, islets are placed in a chamber and an effluent is mixed with anti-insulin antibody and fluorescein isothiocyanate-labeled insulin (FITC-insulin). Insulin from the islets competes with FITC-insulin for binding sites on the antibody [96]. Then, bound, and unbound FITC-insulin are separated in the electrophoresis channel, and insulin secretion is then quantified by establishing the ratio between bound and free insulin. The Kennedy group pioneered the development of a microfluidic CEI to monitor online the fast kinetics of hormone secretion from a single islet with high temporal resolution [95,96,97,98]. This technology can be fully integrated on a chip. Advantages of using CEI include fast and accurate assessments and have proven to detect secretion changes within seconds. However, CEI has been shown to be unsuitable for continuous monitoring of living cells [96]. Using a similar technique is possible to identify glucagon, but this is more challenging since pancreatic alpha-cells comprise a smaller proportion of the islet than β-cells [95]. Likewise, the use of this technique has been extended to monitor insulin and islet amyloid polypeptide (IAPP) secretion profiles at the same time [99]. It is worth mentioning that IAPP is a hormone co-secreted with insulin from islet β-cells in response to nutritional stimuli and acts as glucose regulators in a coordinated manner. IAPP monitoring is of particular interest since it has been considered that aggregation of human IAPP into organized deposits to be a pathological characteristic of DM, contributing to β-cell dysfunction and death and leading to islet transplantation failure [91,100]. However, islet transplantation success requirements are multiple and complex.

In a different approach, several microfluidic chips incorporated intracellular fluorescent detection of Ca^2+^ and mitochondrial activity to determine islet cell physiological behavior [91]. By visualizing calcium oscillations that precede insulin exocytosis it is possible to study mechanisms of secretion and indirectly measure the islet secretion. Therefore, the kinetics of insulin secretion is determined by changes in Ca^2+^ and mitochondrial activity in β-cells [101]. This is, however, not without limitations in terms of quantification and selectivity. It can be challenging to quantify calcium oscillations because of the difficulty in distinguishing these oscillations in different types of cells. Also, it often requires performing traditional immunoassays like ELISA (enzyme-linked immunosorbent assay) to quantify, off-chip, the secretion products. Nevertheless, this technique has led to advances in understanding significant aspects of islet secretions, such as the limited coordination of Ca^2+^ oscillations in islets when stimulated with glucose [102].

There are other examples of MPS specifically designed for pre-assessment of islets destined for transplantation. In this regard is worth mention the work developed by Adewola and Mohammed et al. [103,104], where a MPS device was created to assess the dynamic insulin secretion from multiple pancreatic islets with simultaneous fluorescence imaging of Ca^2+^ oscillations and mitochondrial potential changes; Silva, et al. [105] designed an islet trapping device that allows fluids to bypass the islets through connected channels, thus reducing shear stress on the islet and enhancing β-cell and endothelial cell preservation.

Even though some of the mentioned designs can perform multi-parametric islet characterizations, most of them can only assess a single parameter of secretion and functionality. Focusing only on a single hormone secretion, mainly insulin or glucagon, offers an incomplete outline of islet physiology. This intensifies the need for a multimodal physiological functionality assessment tool to expand the number of hormones detected to measure a greater islet secretory fingerprint (SF).

This need for an accurate SF in an in vivo-recreated microenvironment, provided by MPS systems, is of particular interest in enabling a comprehensive drug screening platform for the discovery of novel therapeutic agents for the treatment of diabetes.

**Figure 4:**
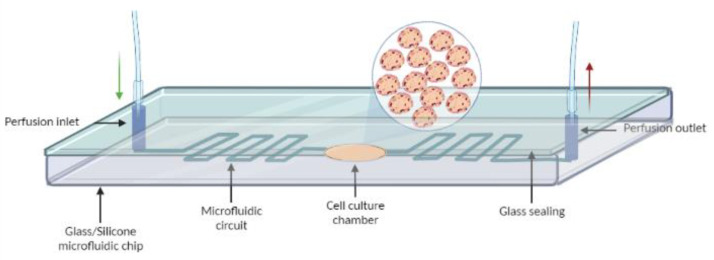
Basic concept of a microfluidic platform (Microfluidic perfusion systems, MPS). A glass/silicon structure containing a 3D microfluidic circuit that move or analyze a tiny volume of liquid, a cell culture chamber or microfluidic chip and a PDMS (polydimethylsiloxane)/glass sealing structure that closes the microfluidic circuit. The perfusion system includes a pump connected to allow a continuous inflow of nutrients and the outflow of metabolites. Created with BioRender.com.

Summing to the precision and control over experiments, MPS can be conducted at a faster pace and lower cost than conventional 2D or 3D cultures. A simplified schematics of a basic MPS is illustrated in Figure 4.

In fact, microfluidics technology is rapidly evolving into different research branches targeting different segments of islet physiology. Some researchers are work in MFS to study T1DM pathways, some are studying encapsulation techniques for transplantation, some are testing cell coatings, others PEGylation or insulinotropic factors, others are looking into the vascularization of cells and/or islet organoids. This proves the multidimensional potential of this technology, in the biomedical field alone, however it has limitations. The use of nanotechnology and microdevices for T1DM therapies has been reviewed by some authors such as Ernst et al. [106], Lai et al. [107] (modular microfluidics) or Lui et al. [108] (paper-based microfluidics for glucose detection which can even be used as a telemedicine device for physicians insulin control off-site).

### 3.3. Potential Analytical Tools for Islet Secretory Fingerprint (SF) Analysis

**Figure 5:**
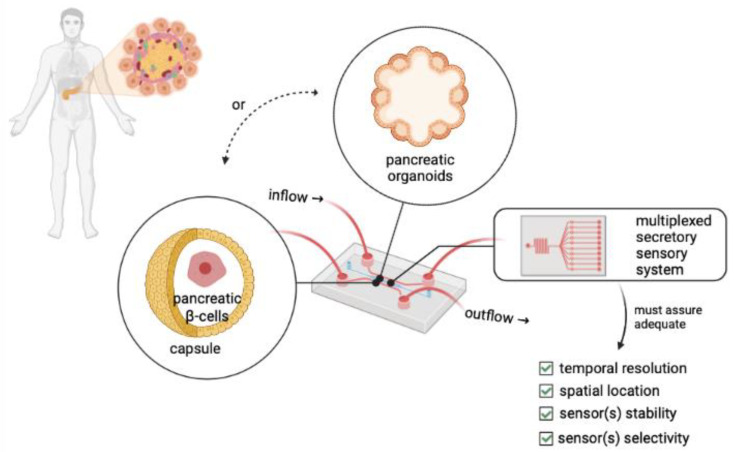
Islet secretory fingerprint analysis is essential in models of insulin secretion and pancreatic function. One of the most important components of a MPS is the sensory system. To assure the fit and quality of the model, the sensors must be carefully located and be highly selective and sensible to retrieve information in real-time of ion channel activity, gene expression, and physiological changes such as insulin production. Cells can come from the patient, making of it a personalized in vitro model, i.e., a specific model that can assess beforehand the best therapeutic option for a person or, for example, the specifics of T1DM microenvironment in a person. Created with BioRender.com.

As mentioned in the previous section, several analytical techniques have proven to be suitable for evaluating the dynamics of insulin secretion. Nonetheless, to reach a more relevant characterization of islet physiology, an integration of different multiplexed detection tools into MPS is needed (Figure 5). Key considerations for detecting multimodal secretions from islets are (a) the temporal resolution of the sensor to guarantee the detection of the fast dynamics of islet hormonal secretion, (b) the sensor dimensions to assure that it can spatially locate islet secretion, (c) the sensor stability to allow continuous operation and, (d) the selectivity of the sensor for a single secreted biomolecule and not a co-secreted product [104]. Ideally, this monitoring tool must be fast, specific, and have high spatio-temporal resolution to be able to assess changes in different islet cells at the same time. Also, these tools need to be modelled to be easily integrated with in vitro or ex vivo cultures via MPS systems.

Some of these microfluidic perfusion systems (MPSs) for secretion fingerprint analysis of pancreatic islets have been reviewed by others [92,109]. Researchers continue to improve cell culture methods in combination with MPSs and cell secretion analysis tools to better create a microfluidics network with sensors that can find applications in determining islet or pancreatic organoids quality, islet regeneration, and drug screening [93,110].

**Figure 6:**
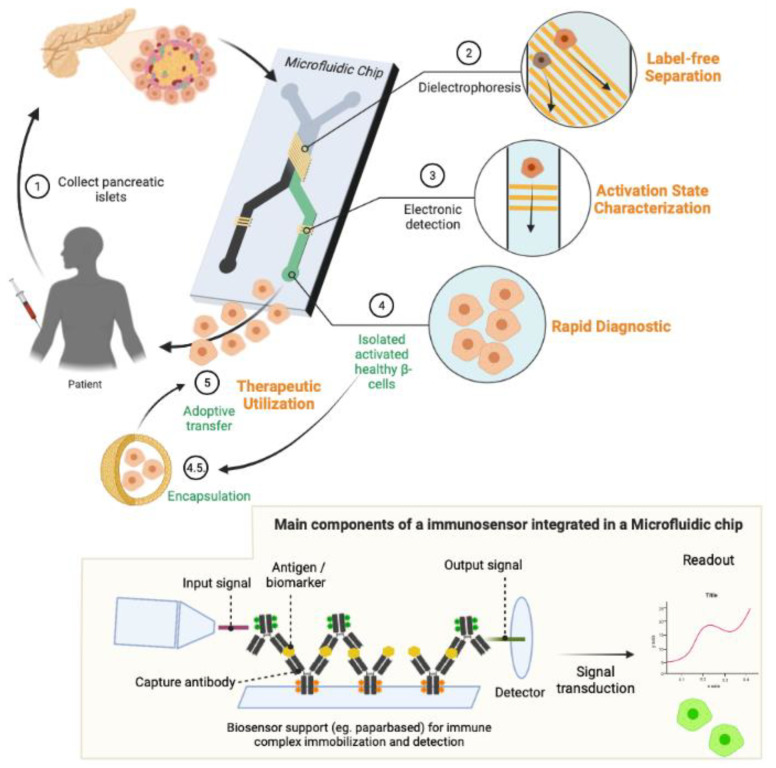
Example of a microfluidic device label-free electrical/immuno biosensors for islet regeneration and/or assessment. A label-free biosensor can be an immunosensor that follow a typical sandwich immunoassay; where a device is capable of immobilizing or target a specific molecule or molecules combination for detection and allow the conversion of an input signal to a quantifiable output signal. Please see, for example, Lara and Perez-Potti, 2018 [124]. Created with BioRender.com.

**Figure 7:**
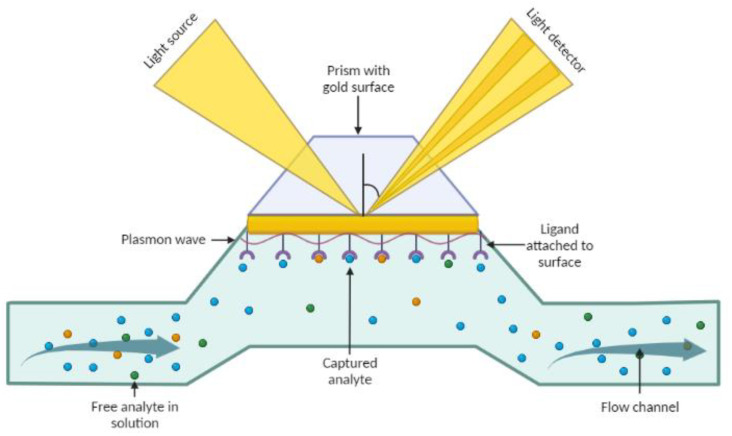
Surface Plasmon Resonance biosensor for multiplexed detection of cell secretion byproducts. Plasmon waves are sensitive to the refractive index of the medium near the surface through which they travel. Taking advantage of this property, the SPR monitors molecular interactions without the need to label any reagent. One binding partner (ligand) is immobilized on the surface of the thin metal layer and the other binding partner, the analyte, flows towards them. As complexes form in the binding areas, the accumulation of mass on the surface changes the refractive index, which can be monitored in real time by the SPR detector. Created with BioRender.com.

Some techniques reportedly promise to deliver a multiplexed detection tools for islet secretions, thanks to the progress of micro and nanotechnologies for the development of electrochemical-, electrical-, and optical-based biosensors [111]. Among these technologies, label-free electrical sensors based on interdigitated electrodes (IDEs) (Figure 6) and optical sensors using surface plasmon resonance (SPR)—Figure 7—have shown great potential [110,112]. However, the challenge to recreate the complex pancreatic T1DM microenvironment remains.

#### 3.3.1. Label-Free Electrical Biosensors

Label-free biosensors generally use a transducer to convert the stimulus-induced cellular response into a quantifiable signal, that is, converts biological signals into electrical impedance signals, that is, it detects whole biologically active molecule in real time [113,114,115,116]. Essentially, capacitance biosensors based on interdigitated electrodes (IDEs) analyze the changes in resistance and capacitance signals at the electrode surface to observe the adsorption and interaction of the biomolecules on the electrode surface [117]. This type of thin film electrodes offers the possibility of fabrication with common microfabrication techniques and adjustable performance by modifying their dimension [118]. Additionally, as a label-free technique, it eliminates costly sample preparation steps, presents instrumental simplicity compared to other techniques, provides a large sensing surface and a possibility of MPS integration that can achieve a multiplex detection of analytes [119]. Here, it can be applied, for example, for cell separation upon biopsy or stem cell culture—(Figure 6). The use of this method to create label-free biosensors has already been explored, for example against infections such as dengue or Zika [120] but more notably for glucose or albumin monitoring [121,122]. Recently, Yoo et al. (2021) [123] described an interdigitated electrode biosensor based on plasma deposited TiO_2_ nanoparticles for detecting DNA. They successfully used a DNA probe and the target DNA to select specific nucleotides and thus, detect certain pathogens such as *Escherichia coli* or *Salmonella* spp. This could also be applied in T1DM using specific genetic or protein markers in transmembrane channels, for example. Nonetheless, research on microelectronic devices to improve sensitivity, specificity, time of detection and real-time multiplex assessment, need further development. Nanoparticles is another parallel field, fast developing, and that if used for T1DM research and to recuperate or recreate islets and/or T1DM microenvironment needs to be fully adequate and non-toxic to use with live animal cells.

#### 3.3.2. Surface Plasmon Resonance Imaging (SPRi)

Surface plasmon resonance, SPR, biosensing has become the gold standard to study biomolecular interactions, especially affinity-based interactions such as antigen-antibody [110]. SPR, in essence, allows fast detection of binding interactions due to changes in the surface plasmon of a thin gold film (Figure 7).

This method not only provides dynamic, label-free and real-time analysis, but also offers the possibility of high-throughput multiplexed analysis by making arrays of different molecules on the sensing surface [110]. SPR biosensors have been used over the past decade to explore fundamental physiological aspects of various islet hormones, namely insulin [125], somatostatin [126] and pancreatic polypeptide (PP) [127]. Still, none of these reports exploited the multiplex capabilities of SPRi by monitoring more than two of the major secreted hormones. As a label-free biosensor, this technique struggles with specificity, especially in environments that require complex matrix, such as a pancreatic cell secretome.

#### 3.3.3. On-Chip Applications of Islet Secretory Fingerprint (SF) Monitoring

The integration of both platforms, islet-on-a-chip technology, and SF monitoring, can prove to be a new way to study diabetes and β-cell function with high-resolution. On-chip technologies have been designed to mimic the functional microenvironment of the pancreas within a microfabricated device. Most of islet-on-a-chip assays have been based on fluorescence microscopy and mainly used as specialized proof of concept. Combining this technology with more sophisticated platforms with built-in sensors capable of delivering real-time, highly sensitive, and label-free data could result in a powerful tool for the future of diabetes research. These new approaches could mean a comprehensive in vitro 3D solution, capable of laying the bases for modeling the pathophysiology of the disease and screening new drugs and therapies for the treatment of DM [91]. In fact, Patel et al. (2021) [128] recently reported successfully creating an organoid system that preserves pancreatic islet function within a 3D matrix of alginate hydrogel in what they called an Oxy-Chip. They are concerned most with perfusion and hypoxia, as well as β–cell glucolipotoxicity, to create a chip-organoid system that more closely mimics in vivo DM conditions. They isolated islets from rats and islet encapsulation with alginate before combining them into MPSs. But they have proven a concept where the combination of in vitro methods and biosensing can replace traditional PDMS or polycarbonate base fabrication that challenges long-term microfluidic cultures and observe cells viability and conditions in real time, overcoming static cultures’ shortfalls. Nonetheless, cell initial mortality, and even genetic or protein fingerprint, is far from complete. A good MPS model of T1DM should include not only the proper set of cells or 3D tissue constructs (pancreatic organoids), but also different types of sensors such as the following: optical sensors (colometry or surface plasmon resonance, SPR); electrochemical sensors (amperometry, enzymatic, transepithelial/transendothelial electrical resistance, TEER, electrolyte-insulator-semiconductor, EIS); and physical sensors (temperature, pressure, pH, humidity, fluid property). Therefore, fields of engineering and biology need to come together to create the best in vitro model of a tissue or microenvironment as possible, and combining methods and technology is another challenge unto itself.

## 4. In Vitro Research and Future T1DM Related Studies

Most of the current microfluidic platforms is aimed at islet quality assessment for possible future in vivo implantation or diabetes diagnosis, offering encouraging progresses and providing solid bases for forthcoming improvements. Combining emerging technologies of hiPSC or hESC and microphysiological systems could provide a reliable platform for long-term culture of patient specific pancreatic tissue, which could be of great value for diabetes research and drug screening.

In the search for a cure, researchers have also followed other lines and theories to pinpoint the pathological process of T1DM. This includes the identification of the role of exosomes as biomarkers and therapeutic tools for T1DM [129,130]. To highlight here that microfluidics has been developed to isolate exosomes to control autoimmune pancreas inflammation and stop T1DM progression as well as for diagnosis [131].

### Multi-Organ-on-a-Chip

Diabetes is a multifactorial pathology not bound to a single organ, but by the interaction of multiple factors between different organs and tissues, or even different compartments within the same organ. Even though recapitulating individual organ-associated aspects of DM can provide valuable information about the disease, in vitro single-organ models fail to mimic some key physiological dynamics, like endocrine or inter-organ paracrine signaling. Therefore, to faithfully recreate the complete relevant characteristics of DM, models with higher levels of complexity are required.

Few approaches have been documented so far to interconnect pancreatic islets with other cell types. A first approach to modeling a multi-organ MPS platform including a PoC device was published by Bauer et al. [132] in late 2017. By culturing pancreatic islets and liver spheroids in a two-organ co-culture, it was possible to investigate the interaction between them through insulin and glucose monitoring.

Recently, Tao et al. developed a multi-organoid system that explored the interactions in the liver-islet axis in normal and type 2 diabetes [133]. The pancreatic islet-liver axis is closely associated with normal glucose regulation and homeostasis maintenance. In healthy humans, pancreatic islets secrete insulin to promote glucose uptake by the liver from the blood stream. In this study a multi-organ-on-a-chip device was created from hiPSCs-derived cells to simulate the liver-pancreatic islet axis in vitro through a parallel microchannel network connection. The study was able to maintain the perfused co-culture conditions for up to 30 days. It was able to demonstrate a more relevant platform to study diabetes, by assessing organ response to external hyperglycemic stimuli and drugs that are not easily studied in conventional cell culture and animal models. The research team aims to integrate other organs such as brain, muscle, and fat to further reflect the relevant complex physiopathology of DM.

Another study focusing on liver-pancreas dynamics was described by Essaouiba et al. [134] in 2020. Similarly, the study proposed to investigate the close interactions between the two organs and compare their function with hepatocytes (with and without insulin) in monocultures.

The benefits of using co-cultures with two-organ models were demonstrated by the recovery of hepatic function in the co-culture which highlighted several physiological responses. This further validates the potential of multi-organ approaches to investigate complex in vivo patterns using alternative in vitro methods.

Additionally, a pancreas-muscle-liver MPS system was created by Lee et al. [135], where the integration of muscle tissue was possible. The incorporation of muscle is considered important since muscle is one of the key regulators of glucose homeostasis. However, the cells used in this study were of rodent origin, which has an inherent limitation for the construction of a platform that aims to mimic a realistic human model. Although oversimplified and with limits associated with cell origin, the system proved its advantage compared with monoculture systems.

Even though the multi-OoC concept has advanced immensely thanks to the remarkable work of researchers, a “body-on-chip” is still an ambitious dream. Multi-OoC could potentially emulate the entire human physiology offering excellent accuracy and model complexity, which would create a solid base for research some multifactorial pathologies like DM. Advances in stem cell biotechnology open the door to the use of patient-derived cells, tissues or organoids that would reduce the use of animal models that can only provide a flawed prediction for human physiology besides all the ethical and economic considerations. Many technological obstacles still limit the use of multi-OoC, and it is not expected to replace the use of animals in the near future. However, the advantages already showed by multi-OoC at modeling disease make it predictable that more studies integrating multi-OoC may be underway.

## 5. Beta-Cell Replacement (Islet Transplantation)

Pancreatic β-cell replacement offers the potential for physiological glycemic control, avoiding hypo and hyperglycemic episodes. Whole pancreas transplantation is not a viable solution; surgically, it is an aggressive and invasive procedure associated with comorbidities [136]. On the other hand, islet transplantation is considered a relatively safe procedure with less associated comorbidities, making it an attractive therapeutic option for T1DM [54]. Portal vein injection is a common method for islet allotransplantation. The renal capsule has also been studied as a possible transplantation site, as it has a rich blood supply. Still, the availability of islet transplantation as a therapeutic option is severely limited by the scarcity of tissue donors. Using current isolation techniques, only about half or less of the islets present in a pancreas are recovered, making multiple donors necessary for a single transplantation. This is even more exacerbated by the fact that during the islet isolation and preimplantation period, several islets lose their functionality due to physical and oxidative stresses and the deleterious effects of inflammatory cytokines [98]. Culturing islets for 24 to 72 h before the implantation procedure has been adopted to allow the initiation of time-dependent immunosuppressive regimens in the graft recipient and enables quality control testing during this time. Maintaining islet viability remains a challenge as they deteriorate at a fast rate. A donor pancreas contains approximately one million islets, but after purification and culture only about half of this number is successfully isolated [137,138]. As islets are avascular when transplanted, they are susceptible to apoptosis in the liver in the first few days after the procedure. Next, islets are exposed to oxidative stress, inflammation, including instant blood-mediated inflammatory reaction (IBMIR) and rejection from alloimmune and autoimmune mechanism [139]. This leads to a less than 60% of transplanted islets successfully engrafted into the liver [140].

Islet transplantation has consistently improved over the past 20 years, as many enhancements have been made to optimize pre- and post-transplantation procedures. However, it remains a limited and inefficient therapy for the reasons mentioned previously. Additionally, currently prolonged graft survival is achieved by using continuous immunosuppressive drugs, which when used continuously have a toxic effect. Therefore, efforts continue to be made to improve this technique.

### Encapsulation Strategies

Macro or microencapsulation technology has been extensively explored over the last decades. Encapsulation holds the potential to shield islet or stem cells from immune attacks, using a selectively permeable and stable membrane that allows passive diffusion of glucose, insulin, oxygen, and other nutrient exchange, while preventing direct contact with immune cells. Immobilization of endocrine cells into a semi-permeable hydrogel matrix could prevent immune rejection and avoid continuous immunosuppression [141].

In this sense, two approaches in encapsulations were modelled. In macroencapsulation a large mass of islets is encapsulated, usually using hollow fibers or membranes [141]. Major considerations regarding macroencapsulation include the fiber diameter, strength, and stability. Larger diameter fibers limit the diffusion of nutrients which leads to cell death. Smaller diameter fibers, on the other hand, improve nutrient diffusion but make implantation harder as the risk of potential fracture is increased. The main disadvantages of this strategy rely on the poor oxygen diffusion through the fibers, which can compromise islet viability. Also, according to whether there is direct contact with host blood, macroencapsulation can be categorized into intravascular, if the membrane is directly connected to host arteries or extravascular, if the device is not connected to any blood vessel [141]. The main drawback in this technique is the relative low surface-to-volume ratio, which affects oxygen and nutrients diffusion. To counter this and keep an adequate supply of oxygen and nutrients, the islet density inside the macrocapsule is kept quite low (5–10% volume). This makes macroencapsulation implantation almost impracticable because of the large devices that had to be implanted to provide sufficient mass of islets.

In another approach, microencapsulation involves the encapsulation of one or a small number of islets into one semipermeable microcapsule usually measuring less than 1mm [54]. These systems often use a spherical configuration with a higher surface area when compared to the tubular or planar configuration of macrocapsules designs, therefore providing a better diffusion of oxygen and nutrients [142]. Minimally invasive surgery and spherical shapes minimize the immune reactions associated with implantation. Biocompatibility of the materials used is essential to improve the survival rate of the islets [143]. Hydrogels are the most-used materials for microencapsulation systems owing to their good biocompatibility, permeability to oxygen and nutrients, and tissue viscoelasticity. Polymer bio scaffolds are also often used. Microencapsulation concept is promising and has received attention in the last years. Several significant attempts of clinical translation have been carried out. Although promising, microencapsulation viability in humans is still questionable and remains unsuccessful. To mitigate the autoimmune response and subsequent graft loss, encapsulation strategies that simultaneously allow for vascularization and improved biochemical interactions with the microenvironment may need to be developed [144].

## 6. Stem Cell-Based Therapies

Research on T1DM pathogenesis has shown to be a challenge for a few reasons. The retroperitoneal location of the pancreas, combined with the associated risk of pancreatitis, make pancreatic biopsies a challenging and dangerous procedure, causing an understandable shortage of pancreatic tissue samples. The scattered and sparse nature of the insulitic lesions means that multiple tissue samples from one organ are needed for a comprehensive analysis. Laboratorial management of the tissue is a challenge itself because of the high content of pancreatic enzymes, prompting it to autolysis. Moreover, T1DM has a long pre-symptomatic phase, which leads to patients only presenting established disease when most of the beta cell mass has been destroyed, making the study of early stages of the pathogenesis very difficult [145]. Consequently, animal models have been widely used as substitutes of the disease. Nevertheless, as mentioned earlier, animal, and human pathophysiology feature significant differences. While the NOD mouse has been a useful model to study autoimmune diabetes, available human data is bringing to light important differences in pathology between human and rodent disease patterns. It is possible that these differences go some way to explaining why interventions that have been successful in either preventing or reversing the disease process in the NOD mouse have not yielded similar outcomes in human clinical trials [62]. Therefore, there is an urgent need for alternative human models of T1DM disease, which can address species specific aspects of human physiology and allow the study of interventions for disease prevention. This need has therefore paved the way for stem cell-derived in vitro human disease models [146]. In this sense, stem cells have gained attention for their potential as a limitless source of insulin producing β-cell and for holding the promise of playing a key role in future islet transplantation techniques by enhancing their survival and function. Stem cell-based therapies can provide an answer for the limited availability of suitable donors and enhance the success of the transplantation technique.

### 6.1. Stem Cell-Based Approaches

The majority of active and completed trials in the last few years used mesenchymal stem cells (MSC) derived from different origins [147]. Initially, it was thought that generating insulin-producing cells from MSC was a possibility, but clear evidence is still lacking. Instead, current studies aimed at assessing the protection provided by MSC as adjuvants to improve the outcome of islet transplantation [111]. Other approaches have used human embryonic stem cells (hESC) and human induced pluripotent stem cells (hiPSC) to form functional mature insulin-producing β-cells [148,149,150,151,152].Theoretically hESCs and iPSCs maintain their pluripotency after expansion which fulfill the crucial need of unlimited supply of insulin-producing cells for therapeutic purposes. Nonetheless, stem cell differentiation and organoid culture also presents its own challenges and shortfalls. In here, microchips can also assist into establish long-term functional pancreatic islet organoids, for example. The development and challenges of creating a MPS or islet-on-a-chip based on stem cells has been recently discussed by Yin et al. (2022) [153].

#### 6.1.1. Human Embryonic Stem Cells (hESCs)

Human embryonic stem cells (hESCs) are pluripotent cells that differentiate into somatic cells in a developing embryo. Potentially hESCs could be used to generate new β-cells for transplantation purposes of T1DM patients. Studies have identified some molecular cues that mimic stages of β-cell development [154]. After that, researchers were able to differentiate hESCs into pancreatic progenitor, endocrine progenitor, and insulin-producing β-cells by forced expression of pancreatic transcription factors [155,156,157]. These studies provided evidence that hESCs could be used to generate functional cells comparable to mature human insulin-producing β-cells for allogenic treatment of T1DM. However, ethical issues in the use of hESCs are still controversial due to their origin [158]–hESCs cells are derived from 5–7-day old blastocysts and its collection involves the destruction of human embryos. This raises severe controversial questions about the morality of the studies involving hESCs. However, new approaches are exploring the extraction of hESCs or hESC-like cells from the umbilical cord and/or placenta. Nonetheless, the potential associated with this regenerative therapy has led to clinical 1/2 phase trials which already started in the US to evaluate the use of hESC-derived pancreatic progenitors (NCT02239354) [159]. However, there are still critical problems that must be surpassed. Maintenance of homogenous culture conditions is vital to maintain a genetically stable generation of cells, variability in cell survival rate as to be addressed and functional glucose responsive potential of differentiated cells must be better monitored [157]. These, associated with the ethical concerns of using embryo-derived stem-cells, remain the biggest obstacles in hESCs use as a therapeutical option for T1DM treatment.

#### 6.1.2. Human Induced Pluripotent Stem Cells (hiPSCs)

Human induced pluripotent stem cells (hiPSCs) are generated from somatic cells by ectopic overexpression of specific transcription factors. These cells, potentially, have the ability of self-renewal and differentiation, but their genomic stability remains questionable. Nevertheless, hiPSCs biotechnology provides an opportunity to generate patient-specific cell lines that can be differentiated into tissues of interest and then be used for modeling disease pathology or potentially for cell replacement therapy. The hiPSCs have been successfully used to create human models of diabetes caused by monogenic disorders that effect beta cell development and function such as in the Wolfram syndrome [160] and insulin gene mutations [161,162], but only recently researchers started to investigate the potential of hiPSCs in acquired forms of diabetes. These hiPSCs are generated from adult somatic cells that have been reprogrammed back into an embryonic-like pluripotent state using Yamanaka factors [163]. Because of their somatic origin, hiPSCs do not have as much as an ethical concern associated as the hESCs do. This technology can be capable of potentially differentiate an unlimited number of hiPSCs into functional β-cells, delivering an exciting prospect for generating glucose responsive β-cells for transplantation in T1DM patients [159]. Furthermore, the progress of hiPSCs protocols have advanced in a manner that patient-specific hiPSCs can act as an important source of autologous cells for cell-therapy without immune rejection. Although promising, generating β-cells from hiPSCs is a complex procedure which involves forcing expression of transcription factors to mimic normal developmental pancreatic stages [164]. Additionally, there is a widely reported susceptibility of hiPSC-derived graft to teratoma formation [165,166]. Moreover, the costs associated with good manufacturing practices of patient-specific stem cell generation, and their subsequent reprograming, could be astronomical and a real impediment in its general feasibility. Even though arising as a potential alternative to hESCs, hiPSCs have not yet reached the same prominence observed in hESCs protocols for generating mature pancreatic endocrine cells [159]. All these issues remain important obstacles that must be hurdled before clinical translation of hiPSCs-derived β-cells could be a possibility. The generation of pancreatic endocrine cells from hiPSCs could replace allogenic transplantation of islets and it is expected, that in the next decade, many experimental trials for the treatment of diabetes will take place to assess its efficacy and safety.

#### 6.1.3. Mesenchymal Stem Cells (MSCs)

Other approaches involve the use of MSCs as adjuvants in islet transplantation. These cells are multipotent stromal cells with the ability of differentiating into various cell types. They can be obtained from a variety of tissues (Figure 3), with the bone marrow the most used. MSCs are of particular interest due to their anti-inflammatory, immunomodulatory, anti-apoptotic, and pro-angiogenic effects [167]. Also, MSCs reportedly have the potential to enhance islet vascularization and engraftment by stabilizing the vascular network around the graft. Other beneficial aspects shown by MSCs are the paracrine factors that they appear to release, promoting the growth and functionality of neighbor cells. By mixing these beneficial attributes, studies have shown that bone marrow-derived MSCs improve islet graft in rodents [168] by reducing islet apoptosis, increasing the rate of revascularization and better overall islet function, even in microencapsulated islet grafts. Similarly, extenuation of hypoxia-induced damage to the islets was reported. All of these has led to the belief that direct physical contact between islets and MSCs is pivotal for enhancing islet survival.

In another approach, transplantation of adipose tissue-derived MSC with islets demonstrated decreased cell death, better viability, better membrane integrity, and enhanced insulin secretion at glucose stimuli [167,168].

**Figure 8:**
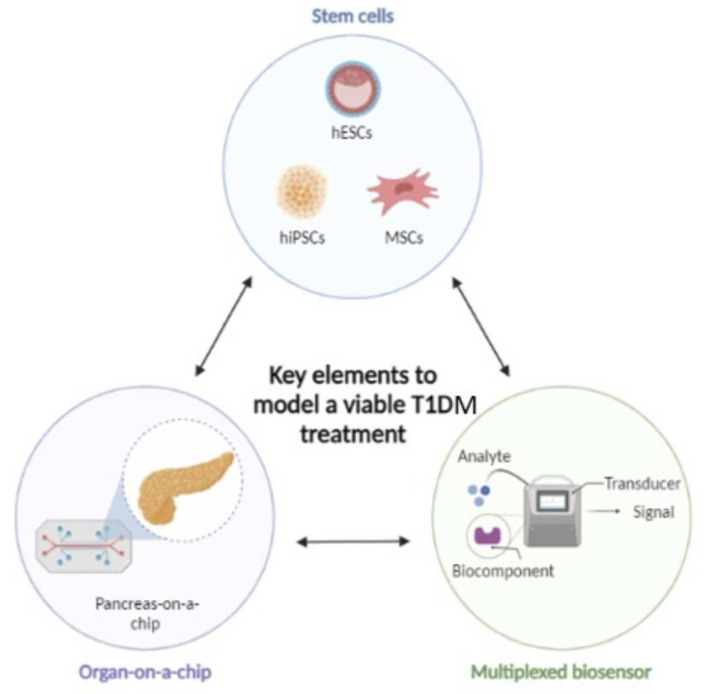
Technologies that can change T1DM treatment approaches. An overview as the triangular vortices relies on each other to model an alternative therapy for exogenous insulin intake. Together, these technologies promise to potentially create mature insulin-producing organoids for transplantation purposes into T1DM patients.

It has been demonstrated that MSCs can have a beneficial effect on the outcome of islet transplantation for treating T1DM, as they enhance engraftment through multiple mechanisms involving MSC-derived molecules. The biggest drawback at the use of this technique relies on the graft site. Clinical islet transplantation occurs almost exclusively via hepatic portal vein which does not facilitate co-engraftment of islets and MSCs. MSCs are much smaller in size than the islets (15–30 μm and 100–200 μm, respectively). Consequently, MSCs can easily cross the hepatic portal system and most likely end-up in lung capillaries [169]. This is an obstacle that must be surpassed to successfully implement clinical practices with islets and MSC co-transplantation, and for which novel microencapsulation methods and biomimetic capsules can offer a solution. Figure 8 demonstrates the potential of technologies to create mature insulin-producing organoids for transplantation in T1DM patients. Table 2 summarizes the advantages and disadvantages of the three main approaches to control or cure T1DM covered in this review.

## 7. Conclusions and Future Perspectives

Type 1 diabetes remains one of the biggest global health concerns in the world. In the last four decades the number of patients diagnosed with T1DM has steadily increased. Complications associated with diabetes culminate in an increased risk of neuropathy, retinopathy, nephropathy, and cardiovascular events. Although enhanced forms of exogenous insulin replacement have improved the quality of life of T1DM patients, through fast- and long-acting insulin analogues, their overall life quality and life expectancy is decreased, hence the enormous need for the development of new approaches for prevention and treatment of DM.

Significant progress has been made since the first attempts at β-cells replacement in humans in the 1960s and 1970s. Transplantation of islets is an exciting prospect for T1DM treatment as it could provide permanent blood glucose regulation and insulin independence. However, this technique is hampered by the limited availability of islets, extensive islet death, poor vascular engraftment, and the need for (sometimes cytotoxic) immunosuppressive treatment. Although already proven effective in some clinical trials, during a relatively long span, these obstacles make islet replacement therapy not yet viable as a treatment option. Therefore, the development of effective techniques for successful therapeutic outcomes is inevitable.

In this regard, to overcome the obvious shortage of human β-cell lines, hESCs and hiPSCs have become notorious in the last decade. Theoretically, hESCs could provide an unlimited source of cells for allogenic transplantation, and clinical trials using hESC-derived islet organoids transplantation have already started. The hiPSCs, on the other hand, have the potential to introduce patient-specific therapies by generating genetically identical cells to the ones of the patient. These would be beneficial in a transplantation scenario because of the autologous character of the procedure: The hiPSC-derived organs can be accepted by the recipient immune system without the need for immunosuppressive therapy. Even though we are still a long way off, β-cells replacement therapies using hESCs and hiPSCs are finally becoming a tangible reality. However, a tremendous amount of clinical testing is required to investigate the various aspects involved in stem cell transplantation, including the long-term safety and treatment viability.

In this sense organ-on-a-chip and multi-organ-on-a-chip concepts become interesting. OoC technology is a promising complement to current pre-clinical models by mimicking the complex pancreatic microenvironment. Multi-OoC are even more significant in the DM panorama, and since it is a multifactorial disease, the integration of higher levels of complexity would be beneficial to correctly model the condition. These microfluidic devices could lay a crucial platform for pathophysiological and pharmacological studies. By exploiting OoC potential, hESC- and hiPSC-derived islets could be investigated in a human-like environment. This would potentially replace animal models, which although useful at providing valuable insight into DM, fail at delivering relevant outcomes when translating the results into humans and the clinical setting. DM has shown to be species-specific; thus, to study stem-cell-derived organoids for the treatment of diabetes, it is crucial to have human-models that combine both human genetic background and increased physiological relevance. Even though it is a recent technology, OoC is imperatively required for hiPSCs and hESCs assessments before a clinical translation could be possible. This leads to a third major condition for a successful novel treatment of T1DM: analytical tools for secretory analysis. If hESCs and hiPSCs can provide unlimited supply of insulin-producing cells for therapeutic purposes and OoC a platform for functionality assessment, it is obvious that a high-sensitive tool for therapy screening would also be required. A real-time, label-free, multiplexed tool would be extremely valuable to monitor the quality and viability of stem cell-derived organoid use. It remains a complex subject, and its feasibility is hampered by technological barriers, but there are already a few devices, such as SPRi, that hold the promise to deliver such tool in a near future.

In conclusion, to date, no models that can completely recapitulate the complexity of T1DM are available and only a few studies have explored stem cell differentiation potential as possible therapeutic solution for T1DM. The successful creation of a hiPSC based T1DM model will allow a more distinct understanding of the disease process and help investigators design better β-cell preservation strategies, like encapsulation systems and MSCs or hiPSCs use. It will be technically and financially difficult and very time-consuming, but the tremendous work already carried out by researchers in the past decade, has shown that an alternative treatment to insulin injections or daily insulin uptake could be feasible in the near future. For this to happen, the use of stem cells, adult or embryonic, OoC platforms, and real-time assessment tools must be closely linked.

## Figures and Tables

**Table 1:** External, environmental conditions such as climatic conditions, Vitamin D deficiency and dairy consumption as potential T1DM triggers.

Dietary Factors, Life Events, Lifestyle, Climatic Condition	Consequence	Ref.
Number of sunshine hours and climate/air temperature.	Lower sunshine hours and lower temperatures could be linked with onset of T1DM.	[30]
Vitamin D deficiency in pregnancy.	Lower levels of calciferol (vitamin D) during the third trimester of pregnancy could be associated with a higher risk of developing diabetes.	[31]
Sun exposure/Vitamin D deficiency.	Higher sun exposure associates with lower risk of developing T1DM.	[32]
Intensity of UVB radiation and the number of sunshine hours per day.	Higher UV-B exposure in the third trimester and first year of life appears to interact with lower T1DM associated risk.	[33,34]
Association between environmental factors and risk of T1DM.	Maternal age > 35, duration of > 6 months of cow milk feeding, and cesarean delivery were associated with T1DM.	[35]
Seasonal variation of T1DM.	T1DM diagnosis show a predominance in the cold months of the year.	[36]

**Table 2:** Overview of the current three more common T1DM therapies. Shaded cells represent disadvantages of the respective therapeutic approach and no shading marks advantages.

Insulin Injection	Pancreatic Islets Transplant	Stem Cell Therapy
Lifesaving	Improved glycemic control	Virtually illimited source of insulin-producing cells
Decreased quality of life	Release dependence from exogenous insulin	Can help to improve transplant techniques success (e.g., MSCs, encapsulation)
Expensive	Mostly, safe	Autologous transplant (no immune rejection risk; hiPSCs)
Not accurate	Limited number of available viable cells for transplant	Precision medicine with primary cell line cultures directly from the patient’s own cells (hiPSCs)
Associated comorbidities	High rates of transplanted cell death	Ethical questions (hESCs)
	Difficult vascularization	Difficult cell culture maintenance (hESCs & hiPSCs)
	May require immunosuppression treatments (cytotoxic)	Requires new methods to improve glycose stimuli monitoring
		Formation of teratomas
		Very expensive
		Requires the development of platforms for functionality and safety assessments

## Data Availability

Not applicable.
